# Mesenchymal Stem Cell Transplantation for the Treatment of Age-Related Musculoskeletal Frailty

**DOI:** 10.3390/ijms221910542

**Published:** 2021-09-29

**Authors:** Elancheleyen Mahindran, Jia Xian Law, Min Hwei Ng, Fazlina Nordin

**Affiliations:** Centre for Tissue Engineering and Regenerative Medicine, Faculty of Medicine, Universiti Kebangsaan Malaysia Medical Centre, Jalan Yaacob Latif, Kuala Lumpur 56000, Cheras, Malaysia; elan23.97@gmail.com (E.M.); lawjx@ppukm.ukm.edu.my (J.X.L.); angela@ppukm.ukm.edu.my (M.H.N.)

**Keywords:** mesenchymal stem cells, aging, frailty, musculoskeletal system, bone, muscle

## Abstract

Projected life expectancy continues to grow worldwide owing to the advancement of new treatments and technologies leading to rapid growth of geriatric population. Thus, age-associated diseases especially in the musculoskeletal system are becoming more common. Loss of bone (osteoporosis) and muscle (sarcopenia) mass are conditions whose prevalence is increasing because of the change in population distribution in the world towards an older mean age. The deterioration in the bone and muscle functions can cause severe disability and seriously affects the patients’ quality of life. Currently, there is no treatment to prevent and reverse age-related musculoskeletal frailty. Existing interventions are mainly to slow down and control the signs and symptoms. Mesenchymal stem cell (MSC) transplantation is a promising approach to attenuate age-related musculoskeletal frailty. This review compiles the present knowledge of the causes and changes of the musculoskeletal frailty and the potential of MSC transplantation as a regenerative therapy for age-related musculoskeletal frailty.

## 1. Introduction

The rising number and proportion of aged people in the population is a global demographic trend. According to the United Nations, the proportion of the world’s population aged 65 years old and above in 2019 is expected to double the numbers by 2050, reaching over 1.5 billion elderly worldwide [1]. As the geriatric population increases across the world, age-related frailty is becoming a growing public health problem internationally, particularly in countries with highest longevity [2]. Frailty is defined by an age-related decline in functional reserve of multiple body systems leading to a reduced ability to cope with acute or external stressors and is characterized by easy tiring, decreased libido, mood disturbance, enhanced osteoporosis, weakened muscle strength, and susceptibility to disease [3]. As the age of a person advances, the higher they move up in the Clinical Frailty Scale, which translates to illnesses with higher morbidity and mortality [4]. The elderly have a higher risk of developing chronic degenerative diseases such as cardiovascular disease, cognitive impairments, rheumatoid arthritis, and metabolic diseases [5]. The management of the diseases is a heavy burden to the healthcare sector worldwide. 

**Figure 1:**
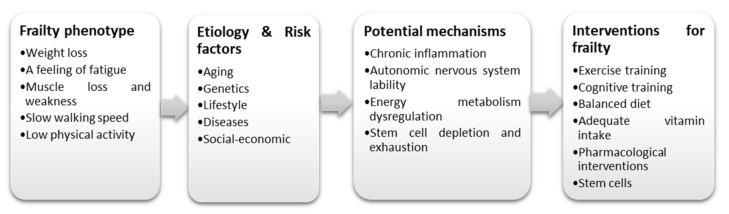
Etiology/risk factors, potential mechanism, frailty phenotype and interventions for frailty.

Meanwhile, musculoskeletal frailty is gaining relevance as a significant clinical syndrome that is associated with increased risk of falls, depression, and disability, leading to higher mortality [6]. Muscle mass and skeletal integrity are both lost as a natural consequence of ageing, starting in the late twenties and accelerating in the fifties [7]. Muscle loss can manifest as age-associated slow loss, termed sarcopenia whereas loss of bone, termed osteoporosis can accumulate to a level where it becomes symptomatic in the form of fractures. In the presence of inherited or environmental factors such as smoking, unhealthy eating, overweight, and physical inactivity, these tissue losses are accelerated [8]. The development of musculoskeletal frailty is also linked with comorbidities such as cardiovascular disease (CVD), endocrine diseases (diabetes mellitus), chronic obstructive pulmonary disease, chronic kidney disease (CKD), anemia, stroke, Parkinson disease, and osteoarthritis [9]. Moreover, previous studies have established associations between chronic inflammation, autonomic nervous system lability, and energy metabolism dysregulation and frailty [10,11]. Figure 1 summarizes the frailty phenotype, risk factors, potential mechanisms, and interventions for frailty.

Many of the health problems of older age are associated with chronic diseases, particularly degenerative diseases and can be prevented or delayed by healthy behaviors. Indeed, physical activity and good nutrition have powerful benefits on health and well-being. Other health problems and declines in capacity can be effectively managed through pharmacological interventions, especially if detected early [3]. Cell-based therapy represents a promising approach to ameliorate and prevent development of frailty since frailty is also associated with stem cell depletion and exhaustion where the stem cell function is characterized by decreased survival, proliferation, differentiation, and homing capacity [12].

Mesenchymal stem cell (MSC) transplantation could be an alternative approach for the maintenance of wellbeing of aged people. A clinical study reported that allogeneic MSC transplantation remarkably improved the physical performance and reduced the serum tumor necrosis factor-alpha (TNF-α) in patients aged between 60 and 95 years in 6 months [13]. Serum TNF-α, C-reactive protein (CRP) and interleukin (IL)-6 are inflammatory markers that are consistently linked with age-related chronic diseases [5]. Numerous studies have reported that MSCs are able to modulate the activities of immune cells which subsequently alter the cytokine secretion [14,15,16]. In addition, MSCs also have been demonstrated to be beneficial in ameliorating degenerative diseases such as cardiovascular disease [17,18], osteoarthritis [19,20], muscular dystrophy [21], and neurodegenerative diseases such as multiple sclerosis [22]. Several mechanisms of action of MSCs have been identified, which are (1) differentiation of MSCs to replace the damaged cells; (2) MSC-damaged cell fusion; (3) paracrine signaling of MSCs which promote tissue repair and immuno-regulate the systemic immune cells; (4) transfer of molecules through MSC-derived extracellular vesicles; (5) transfer of organelles and/or molecules via tunneling nanotubes formation [23]. These findings suggested that MSCs have the capacity to sustain cell growth, maintain viability of tissue cells, and reduce cell apoptosis which are crucial in delaying the ageing-related pathophysiological changes.

## 2. Aging in Muscle

**Figure 2:**
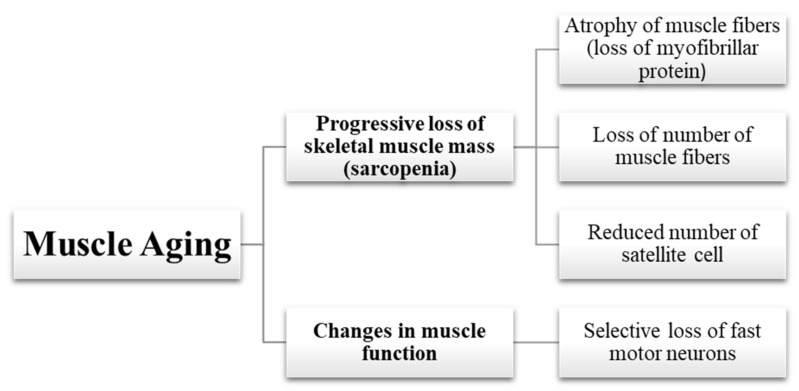
Summary of aging-related pathophysiological changes in muscle.

Muscle aging is linked with a progressive loss of skeletal muscle mass and function. Skeletal muscle is composed of myofibers, which are multinucleated syncytial cells that contain contractile proteins in their cytoplasm. There are two main types of myofibers, classified as slow twitch (type I) and fast-twitch (type II), according to whether they use aerobic (type I) or anaerobic (type II) metabolism. Functionally, aging brings about significant muscle strength loss. Muscle strength can be measured in several ways which are maximum weight moved in a resistance exercise, maximum torque produced eccentrically, isometrically or concentrically, maximum power produced or rate of force development (RFD) where all of these parameters have negative correlation with age [24,25]. Specifically, the ability of the muscle to generate ‘fast strength’ (power or RFD) is severely weakened [26], whereas the ‘slow strength’ is less greatly diminished. On the other hand, muscle endurance or the fatigue resistance of the muscle is not lost to the same extent as muscle strength [7]. All in all, these functional changes can be explained by a few biological changes in the muscle as summarized in Figure 2.

### 2.1. Loss of Muscle Mass

The loss of muscle mass with age can be attributed to atrophy of muscle fibers and loss of muscle fibers. The loss of muscle tissue is low-key during middle age, hovering around 0.5% per year until around the age of 50 years where the loss of muscle tissue shoots up to 1.0% to 1.4% per year [27].

#### 2.1.1. Atrophy of Muscle Fibers

Generally, atrophy of muscle fibers happens due to loss of myofibrillar protein caused by reduced synthesis of myofibrillar and mitochondrial proteins with age [28]. This is especially evident for the fast (type II) fibers that show 15–25% atrophy, which is more prominent in the very fast type IIX fibers than in the type IIA fibers, whereas there is no significant loss in slow (type I) fibers [29,30]. The reduction in myofibrillar and mitochondrial protein synthesis is partly because of the aging associated endocrine changes, especially with the reduced production of anabolic cytokines like insulin-like growth factor 1 (IGF-1) in aging muscle [31]. Aged muscles also demonstrate “anabolic resistance” where it becomes less responsive to anabolic stimuli such as exercise or amino acid consumption that promote protein synthesis [32]. Surprisingly, catabolic stimuli like glucocorticoid hormones also have a reduced effect on aged muscle [33]. It is still unclear which cellular mechanisms are responsible for these altered responses.

#### 2.1.2. Loss of Muscle Fibers

In addition to atrophy of individual fibers, there is also a general reduction in the number of muscle fibers where it has been estimated from cadaver studies that 5% of muscle fibers are lost between the ages of 24 years and 50 years and a drastic 35% reduction is recorded in the next 25 years of age [34]. Fundamentally, these biological changes at the muscle fiber level can be explained in part by the death of motor neurons (denervation) and insufficient reinnervation that lead to atrophy or apoptosis of muscle fibers [35].

#### 2.1.3. Reduced Number of Satellite Cells

Another factor of muscle mass loss is the aging defects in the function of satellite cells. Satellite cells are muscle-committed stem cells located beneath the basal lamina of mature myofibers which plays a vital role for the repair and growth of myofibers. During ongoing muscle regeneration, satellite cells are activated and undergo one or more divisions to form new myofibers or to integrate into existing ones and become myonuclei since skeletal muscle cells are multinucleated syncytia which are formed by fusion of myoblasts. Postnatally, myoblasts are derived from the population of satellite cells. Satellite cells are responsible for skeletal muscle regeneration where they repair damaged muscle and play a role in the maintenance of muscle mass [36]. With age, satellite cell count is reduced up to 50% which leads to a decreased regenerative capacity of muscle [37]. Aged satellite cells have shown reduced activation, proliferation, colony formation, and differentiation in vitro [38]. Jejurikar et al. [39] also had proven that aged satellite cells are also more susceptible to senescence and apoptosis. Moreover, Chakkalakal et al. [40] have demonstrated that with aging, higher levels of fibroblast growth factor 2 (FGF2) is produced by the satellite cell niche which leads to loss of quiescence and self-renewal ability of satellite cells that make them more prone to environmental stresses such as oxidative stress. Satellite cells from aged individuals also undergo differentiation shift where they show increased entry into alternative differentiation pathways, leading to fibroblastic and adipogenic differentiation [38]. Furthermore, elevated numbers of fibro-adipogenic progenitors are discovered in aged muscle [41]. These observations explain the changes in muscle architecture seen with aging, i.e., increased amounts of intramuscular fat deposits and connective tissue.

### 2.2. Changes in Muscle Function

Loss of muscle strength and function happen to an even greater level than the loss of muscle mass and have a significant impact on aged individuals. There are multiple mechanisms behind the loss of muscle function, but the major factor is selective loss of fast muscle fibers due to selective loss of fast motor neurons with aging. Since the type of motor neuron determine the type of muscle fiber, selective loss of fast motor neurons causes the fast muscle fibers to be ‘orphan’ and are then mostly re-innervated by neurons from neighboring slow motor units, leading them to regroup and partially convert to slow fibers, forming a hybrid fiber phenotype or fiber-type switch [34,35]. As a result, normal recruitment of motor units is disrupted and the normal intermixed pattern of muscle fiber types is lost, leading to a decline in motor skills [42]. Moreover, the increase in fibrofatty tissue within skeletal muscle with age also gives rise to the disarrangement and alteration of muscle architecture and thereafter loss of muscle function. In addition, there are intrinsic changes in muscle fibers with aging, such as mitochondrial function defects and increased generation of reactive oxygen species [43] as well as changes in the function and relative amounts of mitochondrial proteins [28], leading to lower respiratory capacity, decreased ATP levels, decreased fatty acid metabolism, intracellular accumulation of lipids, and eventual insulin resistance [44]. Other intrinsic muscle fiber metabolic aging defects include increased glycolysis, decreased glucose uptake and decreased glycogen synthesis [45]. Besides, observations of ultrastructural and molecular changes in aged muscles show that there is defect in calcium storage and release, leading to a decline in calcium homeostasis that affects muscle function [44]. Dystrophin and dysferlin genes which are responsible for membrane repair and stability after tear or destabilization of the muscle membrane due to contraction-induced mechanical stress undergo hypomorphic mutations in old age, which are believed to affect plasma membrane stability [46]. In addition, the decrease in the synthesis of myofibrillar proteins with aging is also influenced by functional changes such as reduced ATPase activity of actomyosin and changes in myosin isoform expression where a knock-on effect of these changes would basically affect the disposition and function of sarcomere-associated signaling factors, causing altered intercellular communication, which possibly lead to an even serious effect on muscle function [44].

Various pathways of the stress response mechanism are activated in response to protein and DNA damage with aging. In a study of aged sarcopenic rats by Altun et al. [47], there is an increased expression of the chaperone-dependent ubiquitin ligase CHIP which catalyzes the degradation of misfolded proteins, and studies with CHIP knockout mouse models done by Min et al. [48] have depicted accelerated muscle mass loss during aging. Although activation of stress response pathways is originally a protective and favorable mechanism, uncontrolled activity may have damaging effects and could ultimately lead to breakdown of not only misfolded or non-functional proteins but also functional myofibrillar proteins, further worsening the protein loss in aged muscles. This can be seen in the effects of the Forkhead box protein O (FOXO) transcription factors, which act as central regulators of the stress response pathways and play a vital role in protein homeostasis where different levels of FOXO activity seem to have distinct and antagonistic effects regarding aging associated changes in protein homeostasis [44,49].

### 2.3. The Muscle-Bone Relationship and Aging

Muscle and bone constitute the unit of motion. Muscles are functionally matched to the bones in size and geometry. When the force generating capability of muscle deteriorates, the anabolic response of bone to muscle-derived stimuli changes, causing changes in the muscle-bone relationship. Data show that exercise can significantly improve bone mass and strength in young animals but not in older ones [50,51]. The possible cause for this change in response is probably due to the reduced osteocyte number and density seen in aged bone, leading to impairment of the signaling network [52]. For instance, muscle contraction from exercising increases myokine secretion which are signaling molecules secreted by skeletal muscles that gives a positive effect on bone formation and an inhibitory effect on osteoclast differentiation while the reduction of muscle protein synthesis and overall muscle function with aging may affect myokine synthesis and secretion [53]. The profile of secreted myokines is also believed to change with aging. Therefore, changes in paracrine signaling are also hypothesized to contribute to alterations in the musculoskeletal unit with aging [52].

## 3. Aging in Bone

Bone aging is associated with bone loss which is a major element of aging frailty. The development of bone aging is quite complex where it involves imbalances not only in the skeletal system but also in the bone tissue locally to a certain degree. There are differences in bone loss systemically and locally, yet the reasons for these differences are still undetermined [54]. The primary causes of bone loss associated with age are still not well studied except for the secondary causes such as loss of gonadocorticoids [55].

Locally, the bone is constantly removed and replaced in a process called bone remodeling [56]. It is a tightly regulated process involving balance between the activity of osteoclasts which play a vital role in bone resorption and osteoblasts which are responsible for bone formation [57]. In the course of time, due to exposure of constant mechanical stress, the bones are susceptible to microcracks and microfractures [58]. This will stimulate the action of bone remodeling in the affected area, where osteoclasts will remove the injured bone tissue, and trigger the recruitment and activation of osteoblasts which lead to bone formation. The dynamic remodeling process is kept in balance in normal healthy adults which means that the removed damaged bone tissue is replaced with new bone tissue [59]. Therefore, the general biological age of the bone tissue is nearly consistent throughout the adulthood of a healthy person [60]. Even so, the rates of bone remodeling greatly differ between the respective bone types, for instance, trabecular bone in the vertebrae is remodeled tremendously and therefore ‘young’ in terms of tissue age, whereas cortical bone is remodeled far slower and thus ‘old’ in terms of tissue age [61].

As the age of a person increases, bone remodeling becomes more inclined towards a slow steady bone loss which is primarily due to loss of bone formation by the osteoblasts [54,55]. Besides, osteoclasts are directly affected by increasing age of bone tissue as a higher level of bone resorption activity is activated in aged bone compared to young bone [62].

Bone tissue age is estimated by evaluating the ratio of alpha to beta isomerized collagen type I (bone resorption marker CTX-I) since its conversion from alpha to beta occurs spontaneously with time and the ratio is associated to bone tissue age [63]. By utilizing this parameter, studies have demonstrated that increased alpha to beta ratio occurs when there is accelerated bone loss, suggesting that age of bone tissue generally decreases on account of the pathology [64,65,66]. However, there is a contradicting discovery that potent antiresorptives like bisphosphonates results in increased bone age, measured using the alpha to beta ratio as well as mineralization density but do not cause increase in bone age in the case of estrogen or selective estrogen receptor modulators (SERMs) [60,67]. The contradiction in the findings is still uncertain but it has been shown that bisphosphonates-induced massive suppression of bone remodeling may cause detrimental consequences on bone integrity [68].

**Figure 3:**
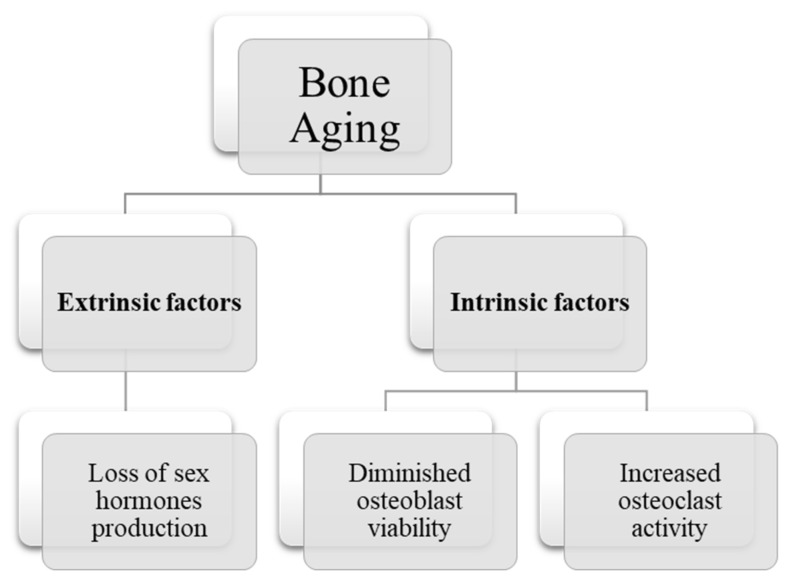
Summary of factors affecting aging-related pathophysiological changes in bone.

There are two types of factors affecting the aging associated changes in the bone as summarized in Figure 3. A few extrinsic factors which are responsible for bone loss with aging have been determined and the primary factor is loss of sex hormone production due to aging [54].

### 3.1. Gonadocorticoids and Age-Associated Hypogonadism

It has been proven that loss of gonadocorticoid production brings about bone loss as a consequence of acceleration of bone turnover [58]. Women are more prone to experience this as there is a strong association between menopause and consequent accelerated bone turnover which lead to bone loss [69]. On the other hand, in men, the consistent loss of testosterone plays a major role in the acceleration of bone loss [70]. Moreover, estrogen levels in men have been proven to be linked to bone density at both trabecular and cortical sites where lower estrogen levels lead to lower bone density which means that bone loss is higher, portraying the protective role of estrogen towards bone in men as well [71,72].

Loss of sex hormone synthesis leads to upregulation of osteoclast generation and bone resorption. At the same time, bone formation is also upregulated due to the coupling between osteoclasts and osteoblasts, but it does not fully compensate for the upregulated bone resorption, and leading to a net decrease in bone mass [59,70]. In women, it has been widely known that estrogen replacement therapy is protective against bone loss, but its long-term effects is comparatively diminished due to the coupling of bone resorption and bone formation [58]. Whereas in men, restoring estrogen levels also mitigates bone loss, similar to the function of restoration of testosterone levels, hence exhibiting the protective effects of gonadocorticoids on bone in both genders [70]. It is hypothesized that the protective effects of testosterone against bone loss in men are mediated by conversion of testosterone to estrogen by the action of aromatase, but its degree of involvement is still being researched. However, the importance of this conversion have been emphasized by studies done in aromatase deficit men [73,74].

### 3.2. Diminished Osteoblast Viability

Bone aging inside associated with certain some intrinsic factors. In a few studies that compared the effects of aging on osteocyte functions, there is a decrease in proliferation, while apoptosis is elevated in aged osteoblast precursors, causing reduction in the potential of bone formation [75]. In addition, bone density is enhanced by transplanting osteoblast precursor MSCs from young to old animals [75], indicating that the bone marrow MSCs partially affect aging-associated pathophysiological changes of the bone. To date, the genetic and epigenetic mechanisms behind these aging defects are still poorly understood.

### 3.3. Increased Osteoclast Activity

Similar studies have shown that when the osteoclast precursor in the blood is expanded ex vivo, the osteoclast viability ex vivo is higher in the old animals compared to the young animals [76,77], exhibiting that age imprinting happens to a certain extent. Besides, data from the study done by Chung et al. [78] indicated an age-related increase in human osteoclastogenesis that is associated with an intrinsic increase in expression of colony-stimulating factor-1 receptor (c-fms) and receptor activator of NF-κB (RANK) in osteoclast progenitors, while in the supporting MSCs, an increase in pro-osteoclastogenic receptor activator of NF-κB ligand (RANKL) expression and a decrease in anti-osteoclastogenic osteoprotegerin (OPG). These findings support the hypothesis that human marrow cells and their products can contribute to skeletal aging by modulating the generation of bone-resorbing osteoclasts, thus showing the potential of MSC transplantation to prevent bone aging.

## 4. Mesenchymal Stem Cells Transplantation for Musculoskeletal Aging Frailty

Although nutritional modulation, cognitive intervention, and physical activity have been shown to ameliorate the onset and progression of frailty or improve the signs and symptoms of frailty, there is currently no proven medical therapy available for the prevention and treatment of frailty [79,80,81,82]. Cell-based therapy presents a hopeful intervention to attenuate and prevent development of frailty. MSC is one of the most promising cell types used in regenerative medicine [83,84].

### 4.1. Mesenchymal Stem Cells (MSCs)

More than half a century ago, Friedenstein et al. [85] described fibroblast-like plastic-adherent stromal cells comprising ~0.01% of the nucleated bone marrow population. After many decades in confusion, in 2006, the International Society for Cellular Therapy proposed minimal criteria for defining MSCs as (a) plastic adherent; (b) expression of CD105, CD73, and CD90 but not CD14/CD11b (monocyte, dendritic cell lineage), CD45 (common lymphocyte), CD79a/CD19/HLA-DR (B lymphocyte lineage), CD34 (hematopoietic lineage); (c) capable of multilineage differentiation [86]. MSCs retain the capacity for post-natal self-renewal and differentiation into multiple lineages, including ectoderm (epithelial and neural cells), mesoderm (connective stromal cells, cartilage cells, fat cells, bone cells), and endoderm (muscle, gut, and lung). MSCs can be identified and expanded from multiple tissues such as peripheral blood, dental pulp, cardiac tissue, adipose tissue, umbilical cord, cord blood, placenta, and bone marrow [87,88,89,90,91]. Although similar, MSCs derived from different sources possess distinct characteristics, advantages and disadvantages, including their differentiation potential and proliferation capacity, which influence their applicability. Hence, they may be used for specific clinical applications in the fields of regenerative medicine and tissue engineering [92].

### 4.2. Mechanism of Actions of MSCs

**Figure 4:**
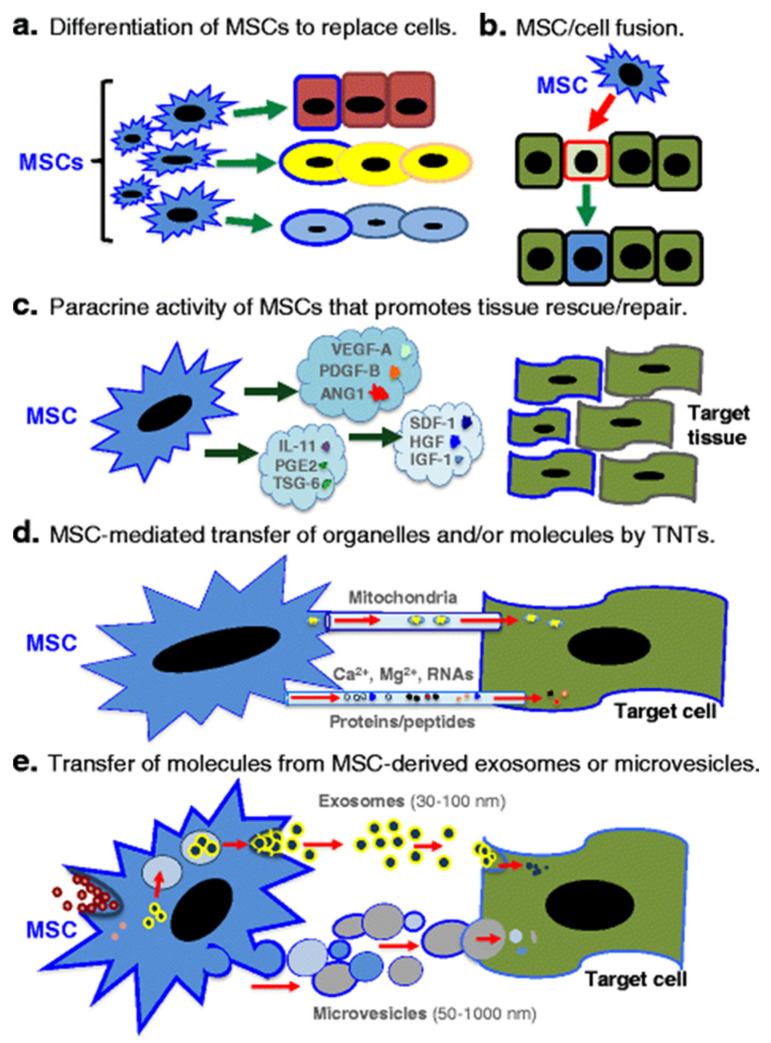
Diverse mechanism of actions of MSCs in rescuing and/or repairing injured cells and tissues. (**a**) Differentiation into replacement cell types. (**b**) Rescue of damaged or dying cells through cell fusion. (**c**) Secretion of paracrine factors such as growth factors, cytokines, and hormones. *VEGF* vascular endothelial growth factor, *PDGF* platelet-derived growth factor, *ANG1* angiopoietin-1, *IL-11* interleukin-11, *PGE2* prostaglandin E2, *TSG-6* TNF-stimulated gene-6, *SDF-1* stromal-derived factor-1, *HGF* hepatocyte growth factor, *IGF-1* insulin-like growth factor-1. (**d**) Transfer of organelles (e.g., mitochondria) and/or molecules through tunneling nanotubes (*TNTs*). *Ca*^2+^ calcium, *Mg*^2+^ magnesium. (**e**) MSC-mediated transfer of proteins/peptides, RNA, hormones, and/or chemicals by extracellular vesicles such as exosomes or microvesicles. Exosomes are generated through the endocytic pathway and released through exocytosis. By contrast, microvesicles are produced by cell surface budding and released directly from the plasma membrane. VEGF: vascular endothelial growth factor, PDGF: platelet-derived growth factor, ANG1: angiopoietin-1, IL-11: interleukin-11, PGE2: prostaglandin E2, TSG-6: TNF-stimulated gene-6, SDF-1: stromal-derived factor-1, HGF: hepatocyte growth factor, IGF-1: insulin-like growth factor-1, Ca^2+^: calcium, Mg^2+^: magnesium. Source: Spees et al. [23].

MSCs migrate throughout the body including the bone and muscle tissues to exert their extraordinary repair and regenerative effects. Other than tissue specific engraftment and differentiation, MSCs are also known to promote tissue repair and regeneration via paracrine signaling (including intercellular communication through extracellular vesicles), mitochondrial transfer and fusion with injured cells as shown in Figure 4 [23].

#### 4.2.1. Immunomodulatory Properties of MSC Facilitate Allogeneic Transplantation

MSCs communicate with both the innate and adaptive immune cells to mediate immunomodulatory and immunosuppressive activities [93,94]. Constitutive expression of major histocompatibility complex (MHC) class I, but not class II, and lack of T-cell costimulatory molecules, e.g., CD40, CD80, CD86, or B7, helps in the escape of MSCs to be captured and destroyed by cytotoxic lymphocytes or natural killer cells [95,96]. Lastly, the most vital part for the therapeutic use of allogeneic transplantation of MSCs is that the allogeneic MSCs do not activate the host lymphocytes, owing partly to MSC secretion of many immunomodulatory factors such as IL-2 and IL-10, interferon-gamma (IFN-γ), TGF-β1, hepatocyte growth factor (HGF), nitric oxide (NO), indoleamine 2,3-dioxygenase (IDO) and prostaglandin E2 (PGE2) [97,98,99].

#### 4.2.2. Exosomes and Extracellular Vesicles

MSC-derived extracellular vesicles (EVs), including exosomes and microvesicles (MV), play a key role in intercellular communication, cell signaling, and altering cell or tissue metabolism at short or long distances in the body. MSC-derived exosomes contain cytokines and growth factors, signaling lipids, mRNAs, and regulatory miRNAs [100,101]. As such, MSC-derived exosomes have great potential as an alternative to whole cell regenerative therapy but this cell-free therapeutic approach for musculoskeletal frailty is still in the early phases. Nakamura et al. [102] reported that purified MSC-derived exosomes accelerated skeletal muscle regeneration in a mouse model of cardiotoxin-induced muscle injury while Choi et al. [103] discovered that exosomes secreted from human skeletal myoblasts enhanced muscle regeneration in a muscle laceration mouse model. However, the signaling molecules involved in skeletal muscle regeneration still remains unclear. On the other hand, the role of MSC-derived exosomes in bone fracture healing was firstly described by Furuta et al. [104] in 2016. Similar promising results were obtained by Qi et al. [105] for the repair of critical-sized bone defects through enhanced angiogenesis and osteogenesis in osteoporotic rats as well as in a study conducted by Yang et al. [106] where MSC-derived exosomes have been shown to prevent disuse osteoporosis by inhibiting apoptosis via miRNA pathway.

#### 4.2.3. Mitochondrial Transfer

MSCs can interact and connect to target cells such as skeletal muscle cells via formation of tunneling nanotubes (TNT) for transfer of mitochondria and other organelles. TNTs were first reported in rat pheochromocytoma cells and immune cells as nanotubular highways for intercellular organelle transport [107,108]. Even though MSCs were originally shown to deliver functional mitochondria to tumor cells via this mechanism [109], further work reported transfer of mitochondria from MSCs to other cells as well, i.e., endothelial cells [110], renal tubular cells [111], alveolar epithelial cells [112], and cardiomyocytes [113]. TNTs are 50 to 1500 nm in diameter, tubular structures stretching across up to several hundred microns connecting two cells. TNTs facilitate transfer of various cellular components such as mitochondria, vesicles, endosomes, beta amyloid, viral particles, microRNA, prions, and lysosomes. via the TNT continuity of the plasma membrane and cytoplasm joining the cells [114]. Unfortunately, the formation of TNTs in rejuvenation of the musculoskeletal system of older individuals by allogeneic MSCs transplantation remains unknown.

### 4.3. MSCs to Treat Musculoskeletal Frailty

MSCs have been postulated to be able to ameliorate musculoskeletal frailty and have been transplanted to frail individuals in many studies. MSCs are drawn to sites of injury, where they act to reduce inflammation and promote cellular repair [115]. Notably, MSCs improved physical outcomes of the frail patients by reducing TNF-α and CRP levels and were safe in patients irrespective of age [115,116]. The growing database documenting safety and potential favorable effects of cell-based therapy in frail patients provide justification for the assessment of potential benefits of cell therapy in subjects with frailty (Table 1) [117,118,119]. MSCs secrete paracrine factors, exosomes, and small extracellular vesicles, reduce inflammatory factors, and activate the resident cells after injury [120,121,122]. It has been showed that MSCs could attenuate sarcopenia via increasing skeletal muscle weight and myofiber cross-sectional area in animal models of sarcopenia [123]. The physical performance including muscle strength as well as endurance were significantly enhanced. MSCs also inhibit apoptosis of muscles and suppress expressions of chronic inflammatory cytokines, which may explain the improvement of skeletal muscle strength and function after transplantation of MSCs. In addition, MSCs have capability to activate resident skeletal muscle stem cells, which lead to myogenesis and differentiation of muscle tissues [124]. The positive results provide novel insights into sarcopenia intervention, suggesting a potential role for MSC therapy in aging frailty. For osteoporosis, MSCs from young mice infused into old mice improved age-related osteoporosis and also increased life span [75]. Kiernan et al. [125] reported that unmodified, low-passage MSCs are indeed capable of long-term bone marrow engraftment via systemic transplantation whereas in a study done by Fu et al. [126], MSCs differentiated into osteoblasts and bone formation was induced by inhibition of osteoclast activity. It also has been shown that MSCs promote the proliferation, differentiation, and migration of resident stem cells to prevent cardiomyocyte apoptosis, reducing fibrosis after myocardial infarction by modulating secreted frizzled-related protein 2, IGF-1 hypoxia-induced Akt-regulated stem cell factor [127,128], and the proteins, peptides, and miRNAs secreted in/on exosomes and extracellular vesicles. The outcomes of many CVDs were improved by MSCs, for example, myocardial infarction [129] as well as nonischemic [84] and ischemic cardiomyopathy [130]. It is likely that these beneficial effects are mainly mediated by the secreting function, especially the paracrine system [131], and secondarily by the direct cellular contact, such as the formation of gap junctions through tunneling nanotubes [132,133]. These hypotheses, however, remain to be verified.

Therapeutic effects of stem cells have also been shown in Parkinson’s disease [134], amyotrophic lateral sclerosis [135], chronic obstructive pulmonary disease [136], idiopathic pulmonary interstitial fibrosis [137], diabetes [135], lupus [138], traumatic brain and spinal cord injury [139], stroke [140], and atherosclerosis [141,142]. These indirectly suggest the feasibility of the application of stem cells in frailty treatment. To date, there are two clinical trials that investigated the role of MSCs transplantation in frail older adults as summarized in Table 2. The first study was a phase I open-label trial [143] where allogeneic MSCs collected from the bone marrow of younger donors aged 20–45 years were used to treat 15 frail patients (average age 78 years) using a single infusion of either 50, 100, or 200 million cells. After six months, outcomes that improved included the six-minute walk and TNFα levels, with variable improvements in forced expiratory volume in one second (FEV1), Mini-Mental State Examination (MMSE), and quality of life. No significant adverse effects were recorded, and only one patient developed antibodies that could potentially neutralize the outcomes. The second study by the same group was a phase II randomized, double-blinded trial of allogeneic MSC at two doses (100 or 200 million cells) versus placebo [144]. The participants were 30 frail patients with an average age of 76 years. No therapy-related adverse effects were documented at one month. Improvements were reported for physical performance, the six-minute walk test, short physical performance exam, FEV1, and TNFα mostly in the 100 million cell groups. The authors conclude that the treated groups had “remarkable improvements” in outcomes. There are always caveats associated with interpreting efficacy in small numbers of subjects, yet it is remarkable that a single treatment seems to have generated improvement in key features of frailty that were sustained for many months.

## 5. Limitations and Future Perspectives

MSC therapy involves the injection of a large number of cells. Thus, it may pose safety issues and side effects to the patient. To date, the optimum dosage of MSCs for transplantation is still undefined. Even though studies have reported that the use of dosage as high as 1200 million cells is safe; however, a higher dose does not indicate higher therapeutic efficacy as several studies have reported better clinical outcomes in the lower dosage group. In fact, the minimum dosage that is effective was found to range between 100 and 150 million cells, while doses higher than 200 million were found to be less or not effective [145]. In a phase II clinical study, Tompkins et al. [144] discovered that the therapeutic effects are limited to 100 million cells dosage as there are no significant therapeutic differences between 100 million and 200 million cells in the tested parameters. Another recent clinical trial concluded that administration of 130 million cells is more effective than the lower dosage of 65 million cells in producing immunomodulatory effects in healthy patients [146]. Therefore, it is postulated that a specific range of cell number is needed to exert the therapeutic effects and administration of excessive cells does not provide additional benefits. In the future, studies should be conducted to validate the minimum effective dose of MSCs that can effectively ameliorate musculoskeletal system aging.

Until now, there is no definite evidence that suggests the best source of MSCs for clinical use. The sources are classified into adult and neonatal tissue derived, which have their own advantages and disadvantages. The heterogeneity among MSCs from different sources and the differences in cell treatment protocol make it impossible for direct comparison. Bone marrow-derived MSCs (BM-MSCs) were the default source of MSCs. Nonetheless, the highly invasive procurement procedure, low cell yield (0.001–0.01% of bone marrow mononuclear cells) and multipotency that diminishes with donor age encouraged studies to be conducted on other sources of MSCs. Peripheral blood-derived MSCs (PB-MSCs) mobilized by the G-CSF are identical to BM-MSCs but are more easily procured. However, both BM-MSCs and PB-MSCs have a longer doubling time in vitro compared to MSCs from other sources [92]. PB-MSCs have been reported to possess the highest immunosuppressive capability compared to umbilical cord-derived MSCs (UC-MSCs), adipose tissue-derived MSCs (ASCs) and BM-MSCs [147]. However, contradictory results have been reported in other studies [96]. ASCs can be obtained easily as surgical waste and lipo-aspirates at a high concentration up to 3%, whereas UC-MSCs have the highest degree of multipotency than BM-MSCs and ASCs [147]. Another MSCs that possess similar immunomodulatory properties as ASCs are dental pulp-derived MSCs (DPSCs) which are usually isolated after the surgical removal of wisdom teeth. DPSCs present a high proliferative capacity and easily differentiate into odontoblasts, osteoblasts, and chondrocytes [148]. Due to these characteristics, they have been proposed in regenerative therapies for bone diseases, among other conditions [149].

Next, the optimal method of administering MSCs has yet to be determined. The MSCs can be introduced into the body locally or systemically. Local administration of MSCs targeted the injury site and produced rapid results. However, there is a risk of cell death and bleeding at the site of application [150]. The systemic administration including intraperitoneal (IP), intravascular (IV), subcutaneous (SC), and intramuscular (IM) delivery have varying cell fate and therapeutic efficacy. Castelo-Branco et al. [151] found that the IP method produced better homing and inflammation suppression than IV. On the contrary, Gonçalves et al. [152] contended that the IV administration of MSCs was more effective than the IP method in the treatment of colitis as IV administration managed to stimulate a higher level of immunosuppression. However, MSCs administered through the IV route tend to become entrapped in the lungs, with only 10% of the transplanted cells reaching the site of damage [150,153,154]. Roux et al. [155] stated the preference of IP over IV as to avoid the risk of pulmonary embolization which may lead to the surge of an anti-inflammatory protein known as TSG6. IM injection is another possible route of MSC delivery which is advocated by Braid et al. [153] for producing the longest cell retention time in the host body when compared to IV, IP, and SC, which was more than 100 days. Both IM and SC implantation sites also retained most of the MSCs, which shows a potential for controlled MSC dosage. Furthermore, IM is less invasive than IV. Nonetheless, the research data on effects of IM administration of MSCs on the musculoskeletal system is inadequate compared to the more established IV method. Ueda et al. [150] injected MSCs contained in collagen scaffold to the dorsum part of mice which considerably prolonged the retention of MSCs at the transplantation site for at least two weeks. The collagen scaffold acted as a reservoir for the exogenous MSCs and preserved the self-renewal, multipotency, and homing functions of MSCs. Furthermore, the formation of aggregates, which commonly occurs with IV administration can be avoided.

Golpanian et al. [143] and Tompkins et al. [144] conducted the phase I and phase II clinical trials in aged patients by administering different doses of allogeneic MSCs through the IV route. The studies monitored the adverse effects as well as the patients’ physical performances and TNF-α level for six months. Both studies demonstrated that 100 million allogeneic MSCs is the most optimum dosage in frail patients which produced significant improvements in both physical conditions. Safety of IV administration of allogeneic MSCs is also demonstrated when treatment emergent-serious adverse events were absent in the treated patients. MSC transplantation is a promising and innovative approach for the treatment of frailty in older humans, and we look forward to the results of phase III clinical trials.

## 6. Conclusions

Aging and ageing-related pathophysiological changes are inevitable. Other than physical activity and good nutrition, there is no effective therapy for ageing-related pathophysiological changes including sarcopenia and osteoporosis. MSC transplantation has been a great promise in attenuating these ageing-related pathophysiological changes. As of now, studies have demonstrated that MSC therapy has great potential in reducing bone and muscle aging frailty. However, its mechanism of action, efficacy and safety are still not fully understood. Therefore, the promise of MSC application in musculoskeletal aging frailty is true, but it requires more time to optimize the culture and processing condition, to elucidate the mechanism of regeneration and repair, and to obtain sufficient safety and efficacy data.

## Figures and Tables

**Table 1:** Potential effects and mechanism of mesenchymal stem cells in patients with frailty.

Frailty Symptoms	Potential MSC Effects	Potential Mechanisms	References
Unintentional weight loss	↓ chronic inflammation	↓ chronic inflammation (↓ TNF-α, ↓ CRP, ↓ IL-1, ↓ IL-6, ↑ TGF-β), ↓ onset of sarcopenia	Jacobs et al. (2013) [119]
A feeling of fatigue	↓ chronic inflammation, ↑ pulmonary function	↓ chronic inflammation (↓ TNF-α, ↓ CRP, ↓ IL-1, ↓ IL-6, ↑ TGF-β), ↑ endothelial function, ↑ pulmonary function (FEV1)	Jacobs et al. (2013) [119]
Muscle loss and weakness	↑ physical activity (six-minute walk distance)	↑ skeletal muscle performance, ↑ cardiac function performance, ↓ onset of sarcopenia, ↑ endothelial function	Fried et al. (2001) [117]
Slow walking speed	↑ physical activity (six-minute walk distance), ↑ pulmonary function	↑ skeletal muscle performance, ↑ cardiac function performance, ↑ pulmonary function (FEV1), ↑ endothelial function	Fried et al. (2001) [117]
Low levels of physical activity	↓ chronic inflammation, ↑ physical activity (six-minute walk distance), ↑ quality of life	↓ chronic inflammation (↓ TNF-α, ↓ CRP, ↓ IL-1, ↓ IL-6, ↑ TGF-β), ↑ skeletal muscle performance, ↑ cognitive status	Jacobs et al. (2013) [119]

Notes: MSCs home to sites of injury and to enhance repair of damaged tissue (heart, joints, muscle, and blood vessels) and exert their regenerative effects via paracrine signaling, mitochondrial transfer, direct cellular contact, and exosome excretion.

**Table 2:** A summary of clinical studies of MSC effects on the musculoskeletal system in frail older individuals.

References	Human Subjects	MSC and Dosage	Results (Related to Musculoskeletal System and Physical Frailty)
Golpanian et al. (2017) [143]	An average age of 78.4 ± 4.7 years and Clinical Frailty Score of 4–6	Group 1 = 20 × 10^6^ allo-hBM-MSCs, IV injection	No treatment-emergent serious adverse events (TE-SAEs) were reported with any of the doses at 1-month.
		Group 2 = 100 × 10^6^ allo-hBM-MSCs, IV injection	Six-min walk distance significantly increased at 3 and 6 months in all treatment groups.
		Group 3 = 200 × 10^6^ allo-hBM-MSCs, IV injection	Physical component of the SF-36 quality of life assessment also showed significant improvements in the 100-million dose group at all time points relative to baseline.
Tompkins et al. (2017) [144]	Age ≥60 and ≤95 years with Clinical Frailty Score of 4–7	Group 1 = 100 × 10^6^ allo-hBM-MSCs, IV injection	No therapy-related TE-SAEs reported at 1-month post-infusion.
		Group 2 = 100 × 10^6^ allo-hBM-MSCs, IV injection	Six-min walk test and short physical performance exam improved significantly in the 100-million dose group but not in the 200-million dose or placebo groups.

## Data Availability

Not applicable.
